# Use of the MANTA Device in Percutaneous Closure of Graft Puncture

**DOI:** 10.1016/j.jscai.2022.100565

**Published:** 2022-12-19

**Authors:** Matthew S. Wu, Shakirat Oyetunji, Creighton W. Don

**Affiliations:** a Department of Medicine, University of Washington Medical Center, Seattle, Washington; b Puget Sound Veterans Administration Medical Center, Seattle, Washington

**Keywords:** graft, percutaneous closure, peripheral artery disease, transcatheter aortic valve replacement

Percutaneous closure for large bore access has been primarily accomplished with suture based vascular closure devices (VCDs), such as the Perclose ProGlide device (Abbott Vascular). Many patients undergoing transcatheter aortic valve replacement (TAVR) present with severe peripheral artery disease and implanted aortofemoral bypass grafts. Femoral access through a graft can be accomplished with small sheaths, but there is minimal literature describing percutaneous closure in synthetic bypass grafts with large bore sheaths. The stiffness of a matured synthetic graft makes puncture with the VCD needles difficult and potentially prohibitive. The recently approved MANTA device (Teleflex) is a passive approximator that uses a collagen plug for closure that could be used in this situation.

The use of larger bore sheaths has been associated with an increasing incidence of bleeding complications from femoral graft puncture^1^^,^^2^ and accounts for increased hospital length of stay and mortality at 1 year.^3^^,^^4^ Thus, the use of percutaneous closure devices in closing bypass graft defects is of great interest to improve patient outcomes. We present a report of the successful use of the MANTA for percutaneous closure of a polytetrafluoroethylene (PTFE) vascular graft in a patient who underwent TAVR.

## Case study

An 88-year-old man with history of severe aortic stenosis and peripheral vascular disease with abdominal aortic aneurysm repair and aortobifemoral bypass using PTFE grafts 20 years ago presented with acute heart failure with indication for TAVR.

**Figure 1:**
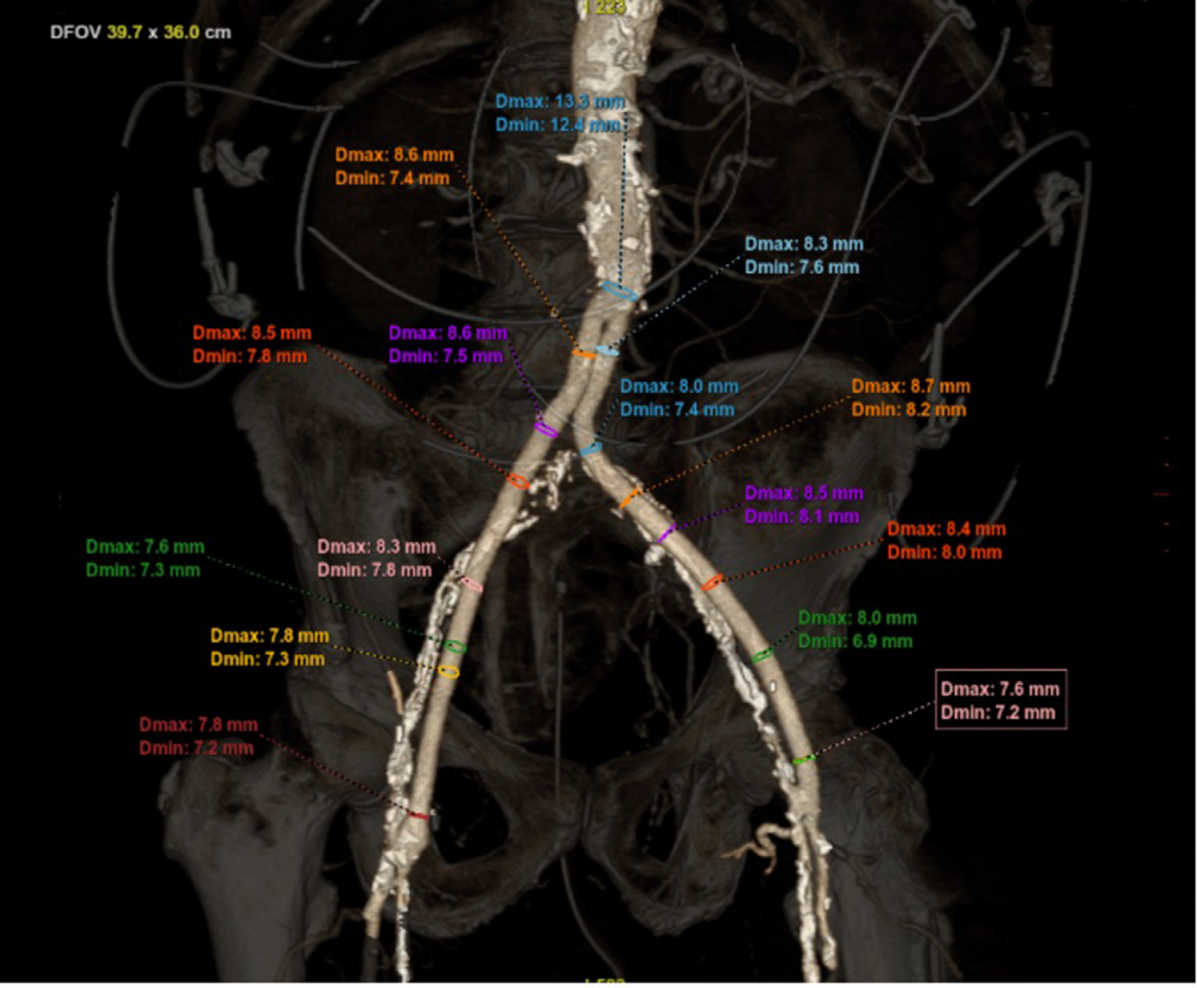
**Preprocedural computed tomography angiography with reconstruction of the iliofemoral vessels, withbilateral aortofemoral bypass grafts demonstrated.** DFOV, display field of view; Dmax, maximum diameter; Dmin, minimum diameter.

His PTFE grafts extended to the distal common femoral arteries, and there was no native femoral vessel available to access (Figure 1). However, a transfemoral approach was still preferred owing to suboptimal alternative access sites (axillary and carotids) that were atherosclerotic and borderline in size and the inherent risk of complications observed in alternative access procedures. The availability of surgical femoral cutdown also provided a bailout option for obtaining access to the graft and if bleeding complications were to arise.

With great difficulty, an 18-gauge needle was able to puncture the stiff, fibrotic graft. The needles from 2 Perclose devices did not initially deploy through the PTFE material. Two more Perclose devices seemed to be delivered, but the plan was made to use a MANTA device for bailout. The depth of the arteriotomy was determined with the MANTA depth locator, and a 14F Edwards eSheath (Edwards Lifesciences) was placed. The TAVR was successfully performed with a 26.0-mm Edwards SAPIEN 3 Ultra valve (Edwards Lifesciences). The eSheath was removed, and the Perclose devices were cinched over a 0.035-inch guide wire but with significant bleeding at the puncture site. Manual pressure was used to obtain temporary hemostasis. The MANTA delivery sheath was advanced without any difficulty and was successfully deployed at the defect in standard fashion, and heparin reversal was obtained with protamine administration. Subsequent peripheral arteriogram demonstrated no contrast extravasation from the puncture site.

The patient was ambulatory within 2 hours without need for further manual pressure, recovered well, and was discharged without incident.

## Discussion

There is a paucity of literature describing the use of VCDs in synthetic peripheral arterial grafts. Vascular surgeons traditionally use manual compression for bypass graft puncture hemostasis for small bore sheaths, which requires at least 10 to 20 minutes of direct compression and 6 to 8 hours of strict bed rest. Even with smaller sheaths, the incidence of complications such as hematomas, pseudoaneurysms, and bleeding is significant. Large bore sheaths, use of anticoagulant agents, and higher patient body mass index increase these risks.^4^^,^^5^ Open vessel cutdowns are often used when deploying large bore sheaths greater than 12F through arterial grafts.

The only randomized control trial that has studied VCD closure of a prosthetic graft was conducted by de Boer et al,^6^ who compared rates of bleeding complications from use of a Perclose ProGlide device with those of manual compression. The rates of hemostasis and bleeding complications were not significantly different, but most of these cases involved access through a biologic patch at the hood of the graft, rather than directly through a PTFE graft (65 vs 8 patients) and evaluated sheaths with a median size of 6F. This study did not include passive approximators such as the MANTA VCD, nor sheaths greater than 14F.

It is unknown whether success of passive approximator VCDs in closing fibrotic grafts is dependent on factors such as graft maturity, material, and degree of fibrosis. Future research is necessary to assess the safety of VCDs in vascular graft closure, especially with attention to graft-specific complications such as inadequate hemostasis and stenosis; however, this successful experience using the MANTA VCD in a patient with a mature PTFE graft demonstrates a proof of concept using passive approximators for closing large bore access sites in very fibrotic synthetic grafts. This represents a potentially useful alternative where suture-based devices cannot be used owing to device needles being unable to puncture the stiff graft material.
